# Updates in biomaterials of bearing surfaces in total hip arthroplasty

**DOI:** 10.1186/s42836-021-00092-6

**Published:** 2021-11-01

**Authors:** Ahmed A. Khalifa, Hatem M. Bakr

**Affiliations:** 1 Orthopaedic Department, Qena Faculty of Medicine and University Hospital, South Valley University, Kilo 6 Qena-Safaga Highway, Qena, 83523 Egypt; 2 Orthopaedic and Traumatology Department, Assiut University Hospital, Assiut, Egypt

**Keywords:** Biomaterials, Total hip, Ceramics, Metals, Polyethylene, Wear

## Abstract

Total hip arthroplasty (THA) is one of the most successful surgical procedures. It entails replacement of the damaged or diseased joint surface with artificial materials. Various materials had been developed and used to achieve optimal outcomes, including longer survivorship and minimal complications. The primary materials used in the manufacture of THA implants are polymers, metal alloys, and ceramics. The failures of THA mainly result from aseptic loosening due to the production of wear particles and the development of periprosthetic joint injection. A lot of advancement and introduction of new biomaterials in THA implants’ armamentarium are designed to avoid the common failure mechanisms and improve the longevity of the implants. In this review, we discussed various aspects of commonly used biomaterials in THA implants, to provide some updated information.

## Background

Since its introduction in the 1960s, total hip arthroplasty (THA) proved to be an effective procedure for managing various hip pathologies, with satisfactory clinical and survival outcomes in up to 90% of the patients in recent 15 to 20 years [1]. In the early 1970s, surgeons were concerned more with problems related to the surgical techniques, structural failure of implants, and infection. However, with the emergence of newer problems that are related mainly to wear and loosening, the concern was shifted and the surgeons’ effort was directed at teaming up with bioengineers to develop new materials and fixation techniques [2, 3]. Nevertheless, it is now evident that long-term THA survival is a multifactorial issue, in which patients, implants and surgeons are all contributing factors and problems with their interaction may lead to various failures [3].

In the past decade, there has been an increasing interest in improving THA clinical outcomes and, at the same time, decreasing the revision rates through improving all procedure-related aspects and developing more efficient surgical techniques (new instruments and emerging approaches). In fact, great attention has been paid to the development of new implant designs and materials by using new biomaterials [4].

LG Donaruma defines a biomaterial as “a synthetic or natural biocompatible material that comprises whole or part of a living structure or a biomedical device which performs, augments or replaces a function that has been lost through disease or injury with no negative effects on the biological environment” [5]. While bioactivity of a material is defined as the ability of a material to bond biologically to the bone, which means that this material may have an effect on or lead to a reaction in the living tissues. For example, ceramics are considered to be an inert material as it does not induce a reaction in the living body [4].

An ideal biomaterial should possess the following characteristics, it should be biocompatible (causing no harm to the host living cells), friction-resistant (having a small friction coefficient which allows for the sliding during repeated cycles of motion with less wear production), corrosion-resistant (can withstand the chemical environment inside the living body), and to have a proper mechanical strength (to withstand the forces and resist early mechanical failures) [4]. In the journey of developing and improving biomaterials used in THA, there have been ups and downs, with the introduction of ultra-high molecular weight polyethylene (UHMWPE) and ceramics being significant achievements. However, some other materials showed catastrophic and disappointing results, like the metal-on-metal (MOM) bearings and the carbon fibers UHMWPE acetabular cups reinforcement [4]. This review aims to shed some light on the development and updates of the bearing surface biomaterials used in THA implants. The issues related to the implants’ designs, geometry, or mechanical properties will not be discussed in this review.

### Why is there always a need for the development of new biomaterials?

Although THA is considered to be one of the most successful surgical procedures performed in the past decade, failures due to several reasons still occur. Understanding the causes of the failure and the underlying mechanisms helps improve the quality of prothesis and materials. For revision THA, the three leading causes of failure include aseptic loosening, infection, and instability [6]. However, a recent systematic review by Kennedy *et al* re-evaluated the causes of failure of revision THA by looking into the literature in the past ten years (involving 9952 revisions). They found that aseptic loosening (which is closely related to implant materials) to be the leading cause for failures, accounting for about 23% [7]. The advances in the development of different biomaterials used in THA will be discussed in the following sections.

Before further discussing different biomaterials and advances related to the wear issue, we first look at the different types of bearing surfaces, which mainly fall into two major categories:

I ***Hard-on-soft bearings:***
  1. Metal-on-polyethylene (MOP):
    A metal femoral head and a polyethylene acetabular liner are the most widely used after their initial introduction by Charnley in the 1970s. It is most widely used in many THA implants for its relative safety and cost-effectiveness [8].
  2. Ceramic-on-polyethene (COP):
    COP consists of a ceramic femoral head and a polyethylene acetabular liner and the ceramic head provides better mechanical properties and high resistance to scratch and deformation. However, it was criticized for its high cost and susceptibility to femoral head fracture [8].
II ***Hard-on-hard bearings:***
  1. Metal-on-Metal (MOM):
    Using MOM was an emerging trend during the late 1990s and rapidly gained popularity among arthroplasty surgeons. They tended to be used for physically-active young patients. Nonetheless, it soon showed catastrophic failures due to metal sensitivity problem and the aseptic lymphocyte-dominated vasculitis associated lesions (ALVAL) [9].
  2. Ceramic-on-ceramic (COC):
    COC was introduced as a hard-on-hard bearing alternative to MOM, to avoid MOM’s major drawbacks as ceramics provided better wear resistance, without metal ion production but with wear particle generation. However, the earlier designs and materials were reportedly associated a catastrophic failure mechanism: either the head or the liner might breaks, and a specific complication, squeaking, was reported in some patients [10].
  3. Ceramic-on-metal (COM):
    With COM, a ceramic femoral head articulates with a metal acetabular liner. COM is designed to avoid the ion production and catastrophic failure of MOM bearing and to prevent fracture and squeaking from occurring with the COC bearing [10]. This bearing couple was supposed to combine the strength of both ceramics and metals [11]. However, a concern was raised about the absolute safety of this bearing couple in a recent prospective, multi-center, randomized, controlled trial by Higgins *et* *al*. When comparing COM with MOM, they found that there was no significant difference between the two groups in the functional and radiological outcomes. Although the chromium ion levels were significantly lower in the COM group after at up to 3 years, it rose at five years. The authors reported some revisions in the COM attributed to metal debris. They concluded that the COM produced metal ions, suggesting there existed a wear pattern different from MOM, and further investigation on its safety and efficacy was needed [9].

To simplify the discussion of advances in the biomaterials of bearing surfaces, we divide these materials into three categories: polymers, metals, and ceramics.

### Polymers

Charnley proposed that polymer materials, with its low friction, should be the first choice for hip arthroplasty. Tests showed that they possessed outstanding mechanical properties and wear resistance. However, in clinical practice, some of these materials showed immune-induced reaction against the wear particles and such reaction led to osteolysis and implant loosening [7].

#### Polytetrafluoroethylene (PTFE)

PTFE is a synthetic fluoropolymer of tetrafluoroethylene, and is thermally stable, hydrophobic, and generally inert in the body. Charnley used it in his first THA trials. However, it showed very high wear rates, as high as 0.5 mm per month with the production of voluminous masses of amorphous materials due to the vast number of foreign-body giant cells [4].

#### Ultra-high molecular weight polyethylene (UHMWPE)

UHMWPE was introduced as an alternative to the failed PTFE after laboratory tests had shown it had promising behaviors, with excellent wear resistance and high strength [4]. In early 1980, the problem of osteolysis and aseptic loosening has surfaced, and the cause was the reaction of the body immune system to the wear particles produced from the UHMWPE. These problems drove scientists to further improve the wear features of the material. In the 1990s, it was proposed that sterilization with gamma irradiation sterilization resulted in the formation of free radicals, which act as precursors of oxidation-induced embrittlement, rendering the material more brittle. This problem was solved by the cross-linking combined with thermal treatment, which produced cross-linked polyethylene (XLPE) with less generation of free radicals and better properties in oxidation and wear resistance and was proved to be effective in clinical studies [4].

#### Cross-linked polyethylene (XLPE)

Although initial results were promising, the cross-linking affected the mechanical properties of UHMWPE, compromising toughness, ultimate mechanical properties, stiffness, and hardness. The cause underlying these shortcomings was the possible formation of free radicals during the manufacturing process, leading to oxidative changes in the XLPE [8].

#### The highly cross-linked polyethylene (HXLPE)

Further improvement on XLPE was achieved by performing cross-linking at a right level of radiation, followed by removing the residual free radicals using two methods: annealing or remelting [12]. The highly cross-linked polyethylene (HXLPE) showed promising clinical results when HXLPE liners were used with delta ceramic femoral head, yielding an annual rate of penetration of 0.022 mm/year, as shown by Kim *et al* [13].

#### Vitamin E-blended polymers

A different approach to stabilize polyethylene (UHMWPE or HXLPE) was recently developed by blending with vitamin E (α-tocopherol) to reduce free radicals production. The rationale behind introducing vitamin E is that it will interrupt the oxidation cycle by decreasing the reactivity of radical species [14]. In a study by Galea* et al* that compared the wear properties of vitamin E-diffused HXLPE with a moderately cross-linked and mechanically annealed UHMWPE in patients in their five postoperative years after THA (involving 221 primary operations). It was found that, five years after primary THA, there was a significantly more penetration of the femoral head in the UHMWPE cohort compared to the vitamin E-diffused HXLPE cohort [15]. There are also some disadvantages with this blend, as for an optimum cross-linking in the presence of vitamin E the concentration should be low; therefore, a balance is needed to obtain cross-linking density and high oxidative stability [14].

#### Polyether-ether-ketone (PEEK)

Polyether-ether-ketone (PEEK) is a biocompatible polymer considered to be an alternative bearing material in THA due to its promising mechanical properties, chemical resistance, inertness, thermal stability, and wear debris biocompatibility [16]. In 1998, Wang and the colleagues tested PEEK acetabular cups on a hip simulator for 10 million cycles and observed significant wear reduction as compared to UHMWPE/ceramic or UHMWPE/metal couples [17]. However, despite the good in vitro data, questions remain regarding the suitability of the material as acetabular cups.

#### Poly 2-methacryloyloxyethyl phosphorylcholine (PMPC)

PMPC is formed by a process known as “photo-induced graft polymerization,” by which the articular surface is covered with a super-lubricious layer that simulates articular cartilage. When the articular surface of XLPE was treated, a chemical thin-layer (100–200 nm) surface cover was formed, and the material showed a dramatic reduction in the wear rate after up to 70 million cycles [18].

#### Polycarbonate-urethane (PCU)

Compliant bearings were introduced as a trial to mimic the mechanical properties of the native articular cartilage. Attempts were made to produce compliant bearings or so-called “cushion bearings”, with proposed optimal lubrication conditions. The research effort was directed at the polyurethane compounds, mainly the PCU, and tribological studies showed that PCU acetabular cups had tremendously low friction when compared to UHMWPE. When it was tested with a cobalt-chromium (CoCr) head in a simulator study of 8 million cycles, it produced wear rates below what had been described with polyethylene cups. However, a concern was raised regarding the amount of creep with further femoral head penetration [19]. In a prospective study by Lazic* et al*, clinical outcomes were assessed after use of the PCU liners. The result with 180 hips exhibited good clinical and survivorship outcomes. Nonetheless, the main drawback of this material, in comparison with metal heads, was hip squeaking [20].

### Metals

A wide variety of metals and metal alloys were used in the manufacture of the acetabular outer shell, the femoral heads and stems [21]. The advances included development of the supporting metals and integration of new metal alloy coatings.

#### Supporting metals and metal alloys

##### Stainless steel

Stainless steels are carbon-based iron alloys that contain Cr, Ni, Mo, Mn, and C (316L). It has the advantage of resistance to oxidation with relatively easier forming, machining, and hardening. However, its use in THA was less popular because of their poor biocompatibility and the high possibility of adhesive and abrasive wear [21].

##### Cobalt-chromium molybdenum (CoCrMo) alloys

CoCrMo alloys are composed of 60–70% Co, 25–30% Cr, 5–7% Mo, with a trace amount of other elements (Mn, Si, Ni, Fe, and C). They can be further divided, in terms of the carbon content, into high-carbon alloys and low-carbon alloys [4]. Although, cobalt and chromium are elements present in food and is necessary to humans as trace elements, they are toxic at high concentrations. Their ions could release into the synovial fluid and then into the bloodstream in patients who received MOM bearing surfaces due to wear of the metal surface. These ions can migrate to the blood before being excreted through the urine and they may affect the biological and cellular functions, making them carcinogenic [14].

##### Titanium alloys (Ti-6Al-4 V)

Ti-6Al-4 V was popular with the manufacturer of the cementless femoral stems and acetabular cups because of its comparatively low density, corrosion resistance, high mechanical strength, and biocompatibility with the bone. However, this alloy is not desirable for the manufacture of femoral heads due to its low wear resistance [21]. It contains vanadium, which is relatively toxic, and attempts were made to replace it with other metals, for example, iron (Fe) or niobium (Nb), leading to the development of improved alloys Ti-5Al-2.5Fe and Ti-6Al-7Nb. These new alloys present superior dynamic hardness and lower modulus of elasticity than the traditional Ti-6Al-4 V and have a better implant/bone stress distribution. Although titanium alloy femoral stems have been used in modular hip arthroplasty since early 2000s, they were recalled by the US Food & Drug Administration (FDA) due to elevated wear debris levels [22]. However, in a recent systematic review by Zajc *et al*, the authors confirmed the shortcomings of using the modular-neck titanium alloy, with no added clinical benefits due to possible worse durability as compared to monolithic stems. However, they suggested that some designs are still being marketed and used worldwide, and the choice to use Ti-6Al-4 V dual modular stem designs during primary THA should be on a selective basis [23].

The shortcomings of the previously mentioned materials led to the development of newer metals with a lower modulus of elasticity and higher resistance to corrosion and wear, *i*.*e*., zirconium (Zr) and tantalum (Ta). They both have excellent chemical stability and an elevated melting point with the added advantage of being highly resistant to corrosion. These properties are ascribed to the stability of the oxide layer [21].

##### Zirconium alloy (Zr-2.5Nb)

A relatively new zirconium alloy (Zr-2.5Nb) was used for the THA bearing surfaces in 2003 in the form of an oxide ceramic layer on metal (Oxinium™). It is more than a coating but involves a surface transformation caused by oxygen diffusion hardening process. The surface of the metal zirconium is converted into black zirconium oxide after being heated in an air environment [24]. It is commercially known as Oxinium™ (OxZi; Smith & Nephew, Memphis, TN, USA). It demonstrated increased hardness and decreased surface roughness like ceramics while maintaining high fracture toughness and fatigue strength characteristic of metals [25]. Oxinium™ heads produced about 45% less wear than smooth Co-Cr heads in simulator studies. Even after the heads were roughened, the difference was much more significant in favor of Oxinium heads, with 61% less wear. In a recent metanalysis, Malachias *et al* explored if the Oxinium head offered better clinical results and higher survival rates in patients who underwent THA. They found that OxZi femoral heads had virtually the same wear and survival rates compared with CoCr femoral heads in patients who underwent THA. They recommended against the “routine” use of OxZi femoral heads in primary THAs [25].

#### Metal alloy surface coatings

To improve the hardness and smoothness of the surface in metal alloys and to provide a non-metal surface to avoid metal ion production, various coatings were tested, including “ceramicizing” the femoral head with alumina composites, titanium nitride, diamond and diamond-like carbon, and other methods aiming at changing the composition of the metal alloy [26].

##### Titanium nitride (TiN) coating

TiN is a bioinert ceramic characterized by being hard and scratch-resistant, with a low coefficient of friction. Being tested as a coating with a potential decrease in wear particles due to the improved wear resistance, it also improved the osteointegration, when used as a coating for cementless implants, by increasing the cellular surface for colonization [27]. Recent reviews showed no superiority of TiN to CoCrMo, with some retrieval analyses revealing a breakthrough in TiN-coated femoral heads [28].

##### Silicon nitride (Si3N4) coating

Si3N4 is a bioceramic harder than titanium alloy (Ti-6Al-4 V), with a fracture toughness higher than aluminum oxide, presumably with lower wear rates [29]. It showed better resistance to hydrothermal degradation, higher flexural strength, and behaviors similar to alumina under biocompatibility testing [2]. The surface of Si3N4 showed relatively low immune activation in vitro, and its wear particles exhibited an solubility in blood serum [30]. Studies by Webster *et al* showed that Si3N4 inhibited *Staphylococcus epidermidis* colonization in vivo, suggesting that this material is of bacteriostatic nature [31]. In a recent in vitro wear study, Yorifuji *et al* compared the wear behavior of COP bearing, by using a vitamin-E-diffused cross-linked polyethylene liner and an oxide ceramic, zirconia-toughened alumina femoral head known commercially as BIOLOX®delta, and a new non-oxide ceramic, silicon nitride femoral head against the same liner used with the BIOLOX delta head. An over 5 million cycles of testing showed that the last bearing couple had lower wear rates. They suggested that the tribochemically formed soft silica layer on the Si3N4 heads may contribute to the reduced friction leading to better wear characteristics [32].

##### Diamond-like carbon (DLC) coating

This material was introduced to increase the corrosion-resistance and durability of metals, such as stainless steel, and was characterized by good scratch-resistance, higher hardness, and the possibility of wear minimization when used as a bearing surface. The DLC-coated Ti-6Al-4 V heads against Si3N4 coefficient of friction was lower, as compared to the bare alloy, by up to 150% [33]. However, in earlier clinical studies, DLC showed evident failure, leading to less survivorship when compared to aluminum oxide heads at 8.5 years [34].

##### Aluminum coating

From the notion of integrating the hardness and scratch-resistance of aluminium with the lower fracture risk of metal alloys femoral head, came the idea of producing ceramicized metal femoral heads and acetabular liners (aluminium-coated Ti-6Al-4 V surface) [35]. This coating consists of an outer aluminum oxide layer, serving as a bearing surface, deeper aluminum layer, and an inner aluminum-titanium (Al3Ti) layer between the surface and deep layer. Experiments showed that such composition could achieve improved hardness and better adhesion [35].

##### Nanocrystalline diamond (NCD)

NCD demonstrated biocompatibility in in vitro studies and in a simulator study. Five million testing cycles (being approximately equivalent to five years of use of a THA), showed that Si3N4 bearing surfaces coated with NCD did not develop cracks and delamination, produced no trivial noise production, and had better wear rates as compared to the fourth-generation ceramics [36].

### Ceramics

Ceramic materials were introduced, about 25 years ago, for THA implants. The most commonly used are alumina and zirconia, characterized by good thermomechanical and tribological features, good hardness and resistance to wear [4].

#### Alumina

Aluminum ceramic is considered to be one of the key ceramics used in THA and is commercially known as BIOLOX® (CeramTec GmbH, Plochingen, Germany). Its excellent tribological characteristics, mainly stem from an excellent frictional behavior and a high wear resistance [37]. On the other hand, it has weaker mechanical resistance than other materials. It behaves well under compression but has weak resistance to tensile stresses [11]. Further improvement led to the development of the BIOLOX® forte (CeramTec GmbH, Plochingen, Germany), and its mechanical properties were improved by using smaller-sized grains in raw materials and reducing impurities [10, 11]. The main concern about using these materials was the noise production, or squeaking, and liability to breakage [10].

#### Zirconia

Zirconia has high toughness, good mechanical properties, slightly lower wear rates and outstanding breakage-resistance, making it a better alternative to aluminium for THA [10, 24]. However, concerns were raised regarding the likelihood of zirconia ceramics to undergo monoclinic-phase transformation in vivo, thereby increasing fracture risk and lowering wear properties, after zirconia femoral heads were recalled by one of the main manufacturers in 2001 due to thermal processing-related issues with a specific product [38].

#### Zirconia-toughened alumina (ZTA)

ZTA was a combination of two materials, where zirconia, at 25% in weight, was incorporated into an alumina matrix, to increase toughness [11]. In 2000, this incorporation led to a commercial product known as the BIOLOX® delta (CeramTec GmbH, Plochingen, Germany) [39]. The addition of a fraction of zirconia to alumina results in a composite material that combines the best features of both alumina and zirconia, *i*.*e*., the strength and toughness of zirconia, and the excellent wear resistance and chemical/hydrothermal stability of the alumina [10]. However, conflicting evidence exists regarding the broad application of BIOLOX® delta as a bearing surface of choice. On the one hand, Lim *et al* showed encouraging outcomes and excellent survivorship after 5 years of use of a 32 head or larger BIOLOX®delta CoC bearing. On the other hand, they reported that the risk of noise production remained a real concern [40]. A systematic review by Massin *et al* compared the fracture incidence of the ZTA with that of alumina ceramics, and they found that BIOLOX®delta femoral heads had a reduced fracture incidence of 0.003% against 0.021% with alumina ceramic. However, the liner fracture rate stayed stable at approximately 0.03%. They concluded that fracture could still occur with the BIOLOX®delta under suboptimal implantation conditions, including edge loading, and the precautions for hard-on-hard bearings implantation, such as attention to cup position, and the insertion on morse tapers, still had to be taken with this material [41].

#### Sapphire

Sapphire is a corundum mineral variety comprising 99.99% aluminum oxide (α-Al_2_O_3_) and trace amounts of chromium, titanium, iron, vanadium, and magnesium. Since it physically and mechanically mimics aluminum, it has been proposed as a potential material for bearing surface. The crystals are placed in a vacuum at 2100° Celsius and are then prepared from single-crystal formation [42]. These bearings showed inertness, low friction, high wear resistance, and good biological compatibility. In a small study, five patients received THAs that contained sapphire femoral heads and had good results at 1- and 5-year follow-up [42]. Further clinical testing is needed to validate the widespread use of this bearing surface.

## Possible future innovations

Some appealing technologies and ideas might be involved in the development and improvement of bearing surface biomaterials and couplings. One of the areas of interest is the study on the hip joint lubrication after joint replacement and the role of the synovial fluid in the lubrication of the artificial joint [4]. This led some researchers to suggest production of materials that have heterogeneous and porous surface of the human cartilage, such as nano-hydroxyapatite-reinforced gel [4]. Poly vinyl alcohol (PVA) hydrogels, which is a long-chain hydrophilic polymer, have been proved to be effective after being used to replace the nucleus pulposus in spine surgery and in great toe implants for managing hallux rigidus and were suggested to have potential to be used in hip arthroplasty [43]. Self-healing materials with the ability to automatically repair themselves and to recover their function after being partially degraded or damaged might be seen in the future [44]. Integration of smart technology, which could measure the amount of wear in the bearing surface, is also on the horizon.

## Conclusion

THA is one of the most successful procedures, with a large armamentarium of implants of various biomaterials and designs to choose from. However, the surgeon should avoid what was called the “fashion trade-in THA” and should resist the temptation to use newly introduced biomaterials, as this injudicious usage led to catastrophic failures over the past 60 years with patients being “fashion victims”. Despite the reported successes of currently used biomaterials, the innovation of THA implants is on the rise due to the need to perform surgeries on relatively younger patients seeking higher activity levels. Future improvement will continue to respond to some concerns in the healthcare system, including implant cost reduction while maintaining patient safety, reducing or eliminating metallic components, removing the CoCr alloy and ion production from the equation and lowering the risk of periprosthetic joint infection. Studies on other factors such as patients’ characteristics and surgical precision, as seen in robotic-assisted surgery, should go side by side with the development of biomaterials.

## Data Availability

Not applicable.

## Abbreviations

THA: Total hip arthroplasty
UHMWPE: Ultra-high molecular weight polyethylene
MOM: Metal on metal
MOP: Metal-on-Polyethylene
COP: Ceramic-on-polyethene
COC: Ceramic on Ceramic
COM: Ceramic on metal
PTFE: Polytetrafluoroethylene
XLPE: Cross-linked polyethylene
HXLPE: Highly cross-linked polyethylene
PEEK: Polyether-ether-ketone
PMPC: Poly 2-methacryloyloxyethyl phosphorylcholine
PCU: Polycarbonate-urethane
CoCr: Cobalt-Chromium
CoCrMo: Cobalt-Chromium Molybdenum
Fe: Iron
Nb: Niobium
FDA: Food & Drug Administration
Zr: Zirconium
Ta: Tantalum
OxZi: Oxinium
TiN: Titanium nitride
Si3N4: Silicon nitride
DLC: Diamond-like carbon
Al3Ti: Aluminum-titanium
NCD: Nanocrystalline diamond
ZTA: Zirconia toughened alumina
PVA: Poly vinyl alcohol
